# ATIQCTPC targeting MMP-9: a key step to slowing primary tumor growth and inhibiting metastasis of lewis lung carcinoma *in vivo*

**DOI:** 10.18632/oncotarget.19172

**Published:** 2017-07-10

**Authors:** Yuji Wang, Xinyi Xu, Ce Song, Jianhui Wu, Xi Hu, Haimei Zhu, Xiaoyi Zhang, Yaonan Wang, Lin Gui, Ming Zhao, Shiqi Peng

**Affiliations:** ^1^ Beijing Area Major Laboratory of Peptide and Small Molecular Drugs, Engineering Research Center of Endogenous Prophylactic of Ministry of Education of China, Beijing Laboratory of Biomedical Materials, College of Pharmaceutical Sciences, Capital Medical University, Beijing, China; ^2^ Department of Biomedical Science and Environmental Biology, Kaohsiung Medical University, Kaohsiung, Taiwan; ^3^ Ruikang Hospital, Guangxi University of Chinese Medicine, Nanning, China

**Keywords:** MMP-9, tumor, metastasis, inflammation, TNF-α

## Abstract

In this study we docked (6S)-3-acetyl-4-oxo-N-(2-(3,4,5,6-zetrahydroxytetrahydro-2H-pyran-2-carboxamido)ethyl)-4,6,7,12-tetrahydroindolo[2,3-a]quinolizine-6-carbo-xamide (ATIQCTPC) towards the active site of MMP-9, and showed that ATIQCTPC was able to effectively decrease the level of MMP-9 in the serum and the primary tumor of Lewis lung carcinoma (LLC) implanted C57BL/6 mice. As a MMP-9 inhibitor, ATIQCTPC inhibited the metastasis of LLC, and slowed the growth of the primary tumor of LLC implanted C57BL/6 in mice. The activities of ATIQCTPC to inhibit the ear edema and to decrease the serum levels of TNF-Î± and IL-8 of the mice treated with xylene were explored. The minimal effective dose of ATIQCTPC that can inhibit the primary tumour growth, prevent the metastasis of LLC and reduce the inflammatory response was 0.01 Î¼mol/kg. The minimal effective dose of ATIQCTPC inhibiting tumour growth and metastasis was 100-fold lower than that of (S)-3-acetyl- 4-oxo-4,6,7,12-tetrahydroindolo[2,3-a]quinolizine-6-carboxylic acid (ATIQC, parent compound). The minimal effective dose of ATIQCTPC inhibiting inflammation was 110-fold lower than that of aspirin. These superiorities reflected the rationality of ATIQCTPC design. The safety of the therapy was explained by 1 Î¼mol/kg of ATIQCTPC did not injure the kidney, the liver and the heart of the treated inflammation mice.

## INTRODUCTION

**Figure 1:**
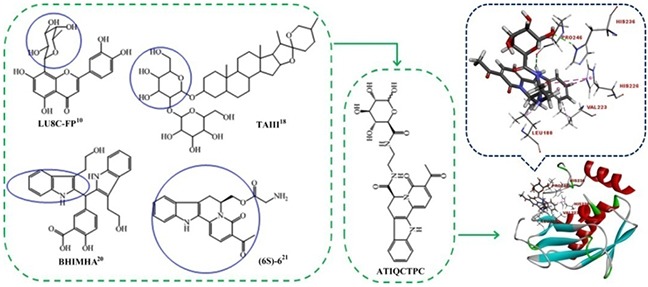
Pharmacophores based design and the active site of MMP-9 based docking of ATIQCTPC

**Figure 2:**
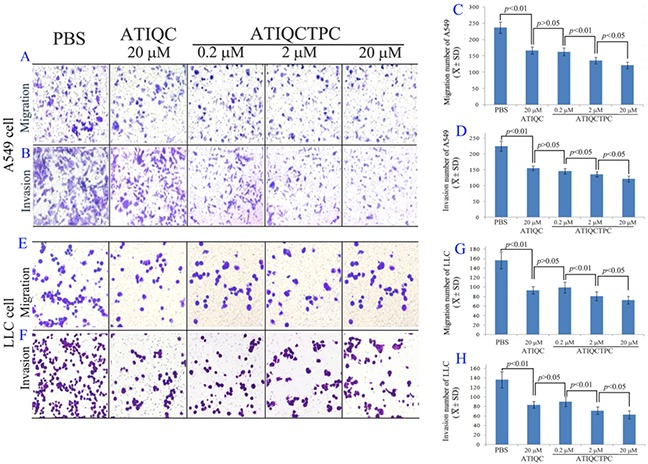
Effect of 0.2 μM, 2 μM and 20 μM of ATIQCTPC on the migration of A549 cells **(A** and **C)**, the invasion of A549 cells **(B** and **D)**, the migration of LLC cells **(E** and **G)**, the invasion of LLC cells **(F** and **H)**, n=12.

**Figure 3:**
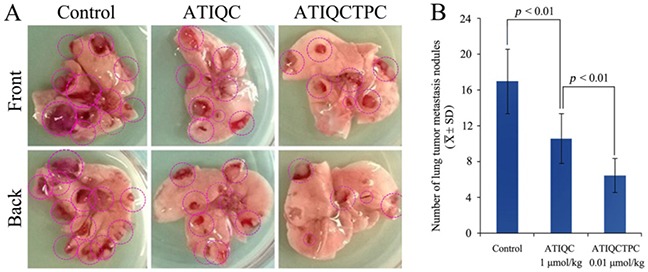
Effect of ATIQCTPC on the number of lung metastasis nodules of LLC sarcoma bearing C57BL/6 mice **(A)** The representative lung of LLC sarcoma bearing C57BL/6 mice orally treated with NS for 11 days (left), the representative lung of LLC sarcoma bearing C57BL/6 mice orally treated with 1 μmol/kg/day of ATIQC for 11 days (middle), and the representative lung of LLC sarcoma bearing C57BL/6 mice orally treated with 0.01 μmol/kg/day of ATIQCTPC for 11 days (right). **(B)** The number of lung metastasis nodules of LLC sarcoma bearing C57BL/6 mice orally treated with NS(control), ATIQC and ATIQCTPC, n=10.

**Figure 4:**
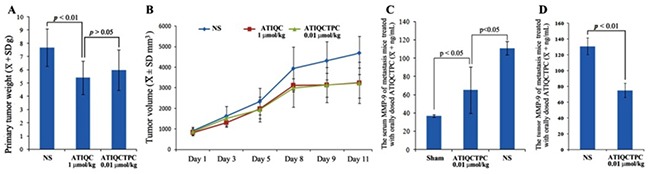
Effects of ATIQCTPC on the primary tumor weight **(A)** and volume **(B)**, the level of MMP-9 in the serum **(C)** and the level of MMP-9 in the primary tumor **(D)** of LLC sarcoma implanted C57BL/6 mice, n=10.

**Figure 5:**
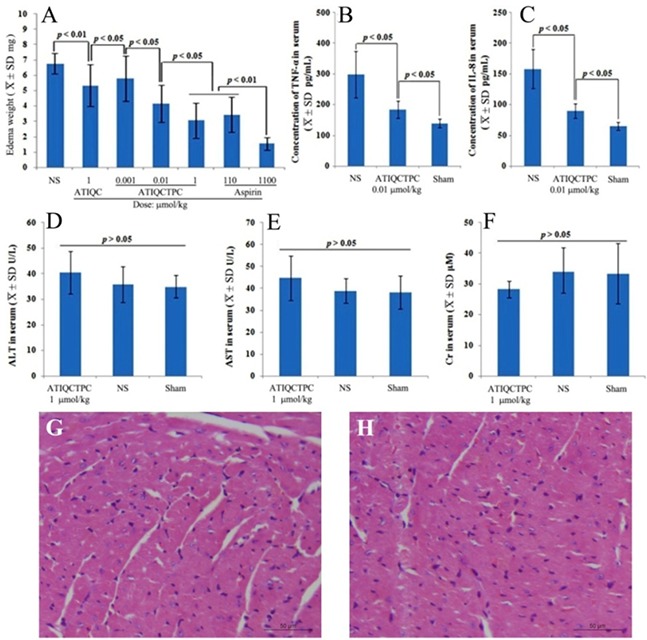
Effects of ATIQCTPC on xylene-induced ear edema **(A)** Dose (0.001, 0.01 and 1 μmol/kg) dependent inhibition of ATIQCTPC to xylene-induced ear edema. **(B and C)** Effect of 0.01 μmol/kg ATIQCTPC on the serum TNF-α and IL-8 of inflammation mice; Effect of 1 μmol/kg ATIQCTPC on the serum AST **(D)**, the serum ALT **(E)** and the serum Cr **(F)** of the inflammatory mice. **(G)** The heart section with H&E stain of the inflammatory mice treated by NS. **(H)** The heart section with H&E stain of the inflammatory mice treated by 1 μmol/kg ATIQCTPC; n=12.

The investigations of matrix metalloproteinase-9 (MMP-9) have attracted the interests of a series of fields. The medical investigations of MMP-9 advance the cancer therapy and the prevention of tumor metastasis. Of the progresses that push the medical investigation forward should be the inhibitors of MMP-9 in targeting therapy. The impact of MMP-9 to tumorigenesis and targeting therapy has been well known [1]. The activation of MMP-9 leads to the migration of breast cancer cells [2]. The relevant expression of MMP-9 in lung tissue induces lung cancer [3]. MMP-9 is considered a potential biomarker of osteosarcoma [4], and is intensely implicated in metastatic progression of colorectal cancer [5]. The inhibition of MMP-9 can effectively attenuate cancer metastasis [6], and at early stages of cancer the inhibition of MMP-9 is essentially efficacy [5]. Usually, the cause of the death of the cancer patients is the metastasis rather than the primary tumor. This emphasizes the importance of inhibiting MMP-9 [5–7]. To prevent the cancer patients from metastatic death, a series of MMP-9 inhibitors such as bioactive polyphenols of green tea [8, 9], naturally occurred products [10–12], clinical drugs [13–16], and the synthetic compounds [17–20] were reported. Since most of these inhibitors received *in vitro* evaluation only, the *in vivo* active inhibitors are urgently needed. In this context, the present paper analyzed the structural characteristics of the above *in vitro* inhibitors, integrated their pharmacophores [10, 18–21], and designed (6S)-3-acetyl-4-oxo-N-(2-(3,4,5,6-tetrahydroxytetrahydro-2H-pyran-2-carboxamido)ethyl)-4,6,7,12-tetrahydroin-dolo[2,3-a]quinolizine-6-carboxamide (ATIQCTPC) as an inhibitor of MMP-9 (Figure 1). The docking assay showed that, of the libdock scores (95.08-118.02) of the 5 compounds in Figure 1 and (2R)-2-[2-[[(2R,3R,4R,5S,6R)-3-acetamido-4,5-diacetyl-oxy-6-(acetyloxymethyl)oxan-2-yl] carbamothioylamino]ethyl-(4-phenyl-phenyl)sulfonylamino]-3-methylbutanoic acid (the standard ligand), ATIQCTPC had the highest score (118.02, see Supplementary Table 2). Figure 1 also shows that the 6 interactions of hydrogen bonds between ATIQCTPC and the side chains of the amino acid residues in the active site of MMP-9 are the major interactions between the standard ligand and the side chains of the amino acid residues in the active site of MMP-9 [22].

## RESULTS

### ATIQCTPC effectively inhibits the migration of A549 cells

The anti-migration activity of ATIQCTPC was evaluated with the *in vitro* migration assay of A549 and LLC cells, and the results are shown in Figure 2A, 2C, 2E and 2G. As seen, ATIQCTPC concentration-dependently inhibit the migration of A549 and LLC cells. The migration number of A549 and LLC cells treated with 0.2 μM ATIQCTPC is significantly lower than that of A549 and LLC cells treated with phosphate-buffered saline (PBS), and equals to that of A549 and LLC cells treated with 20 μM ATIQC. This means that anti-migration activity of ATIQCTPC is 100-fold of ATIQC.

The anti-invasion activity of ATIQCTPC was evaluated with the *in vitro* invasion assay of A549 and LLC cells, and the results are shown in Figure 2B, 2D, 2F and 2H. As seen, ATIQCTPC concentration-dependently inhibit the invasion of A549 and LLC cells. The invasion number of A549 and LLC cells treated with 0.2 μM ATIQCTPC is significantly lower than that of A549 and LLC cells treated with PBS, and equals to that of A549 and LLC cells treat by 20 μM ATIQC. This means that anti-invasion activity of ATIQCTPC is 100-fold of ATIQC.

### ATIQCTPC effectively inhibits the metastasis of LLC toward lung *in vivo*

The anti-metastasis activity of ATIQCTPC was evaluated on Lewis lung carcinoma (LLC) sarcoma implanted C57BL/6 mice, and the results are shown in Figure 3. Figure 3A is the representative lungs of LLC sarcoma bearing C57BL/6 mice orally treated with NS for 11 days, with 1 μmol/kg/day of ATIQC for 11 days and with 0.01 μmol/kg/day of ATIQCTPC for 11 days. On the front and the back of the lungs the metastasis nodules are marked with red rings.

Figure 3B shows the statistical results of the metastasis nodules on the lungs. The number of lung metastasis nodules of the mice orally treated with 0.01 μmol/kg/day of ATIQCTPC for 11 days is significantly lower than that of lung metastasis nodules of the mice orally treated with NS for 11 days, and is equal to that of lung metastasis nodules of the mice orally treated with 1 μmol/kg of ATIQC for 11 days. The data evidence that ATIQCTPC effectively inhibits LLC sarcoma metastasis towards the lung, and its activity is 100-fold of ATIQC.

### ATIQCTPC effectively decreases weight/volume and MMP-9 of LLC sarcoma implanted C57BL/6 mice

The anti-tumor activity of ATIQCTPC was evaluated on LLC sarcoma implanted C57BL/6 mice, and the primary tumor weights and volumes are shown in Figure 4A and 4B. The sarcoma weight/volume of LLC sarcoma implanted C57BL/6 mice orally treated with ATIQCTPC (0.01 μmol/kg/day) for 11 days is significantly lower than that of LLC sarcoma implanted C57BL/6 mice orally treated with NS for 11 days, and is equal to that of LLC sarcoma implanted C57BL/6 mice orally treated with ATIQC (1 μmol/kg/day) for 11 days. The comparison suggests that ATIQCTPC effectively slows LLC sarcoma growth, and its activity is 100 folds of ATIQC. Besides, the efficacy of ATIQCTPC in lowering LLC sarcoma weight is the same as it in limiting LLC sarcoma volume.

Figure 4C shows that the serum MMP-9 level of LLC sarcoma implanted C57BL/6 mice orally treated with ATIQCTPC (0.01 μmol/kg/day) for 11 days is significantly lower than that of LLC sarcoma implanted C57BL/6 mice orally treated with NS for 11 days. While Figure 4D shows that the MMP-9 level in the primary tumor of LLC sarcoma implanted C57BL/6 mice orally treated with ATIQCTPC (0.01 μmol/kg/day) for 11 days is significantly lower than MMP-9 level in the primary tumor of LLC sarcoma implanted C57BL/6 mice orally treated with NS for 11 days. These data suggest that the *in vivo* efficacy of ATIQCTPC (0.01 μmol/kg/day for 11 days) effectively inhibiting the metastasis of LLC toward lung could be the result of it decreasing MMP-9 level in the serum and the primary tumor of LLC sarcoma implanted C57BL/6 mice.

### ATIQCTPC effectively decreases ear edema of the mice treated with xylene

The relationship between MMP-9 expression and inflammatory response was well established [23–25]. This relationship encouraged the present paper to evaluate the anti-inflammation activity of ATIQCTPC on xylene-induced ear edema mouse model, and the ear edema is shown in Figure 5A. ATIQCTPC inhibits ear edema of the mice in a dose (0.001, 0.01 and 1 μmol/kg) dependent manner. The ear edema of the mice orally treated with 0.01 μmol/kg ATIQCTPC is significantly lower than those of the mice orally treated with NS and 1 μmol/kg ATIQC. This comparison suggests that the minimal effective dose of ATIQCTPC in inhibiting inflammation is 0.01 μmol/kg and its activity is 100-fold higher than that of ATIQC. Besides, the ear edema of the mice orally treated with 1 μmol/kg ATIQCTPC is equal to that of the mice orally treated with 110 μmol/kg aspirin. This comparison suggests that the anti-inflammation activity of ATIQCTPC is 110 folds of aspirin.

### ATIQCTPC effectively decreases serum TNF-α and IL-8 of inflammation mice

The relationships between MMP-9, tumor necrosis factor-α (TNF-α), interleukin-8 (IL-8) and inflammatory were widely reported [26–29]. These encouraged this paper to evaluate the serum TNF-α and IL-8 of ATIQCTPC treated inflammatory mice, and the results are shown in Figure 5B and 5C. At a dose of 0.01 μmol/kg ATIQCTPC do effectively decrease the serum levels of TNF-α and IL-8 of the inflammatory mice. These findings suggest that 0.01 μmol/kg ATIQCTPC is capable of simultaneous decrease the serum MMP-9, TNF-α and IL-8 *in vivo*.

### ATIQCTPC does not injure the kidney, the liver and the heart

To estimate the therapeutic toxicity the serum levels of ALT, AST and Cr of the inflammatory mice orally treated with 1 μmol/kg ATIQCTPC were measured, and the data are shown in Figure 5D-5F. As seen, after the treatment the serum levels of ALT, AST and Cr of the mice are not significantly changed. This means that 1 μmol/kg ATIQCTPC does not injure the kidney and the liver. Besides the H&E stain of the heart sections of the inflammatory mice is also performed. Figure 5G and 5H show that the heart sections of the mice treated with NS and ATIQCTPC have the similar myocardium histology. This ensures that ATIQCTPC induces no heart damage. Due to the dose of ATIQCTPC for evaluating serum ALT, AST and Cr and staining heart sections is 110-fold higher than that of its minimal effective dose, the safe window of ATIQCTPC is high enough.

### ATIQCTPC effectively scavenges NO· free radicals

**Figure 6:**
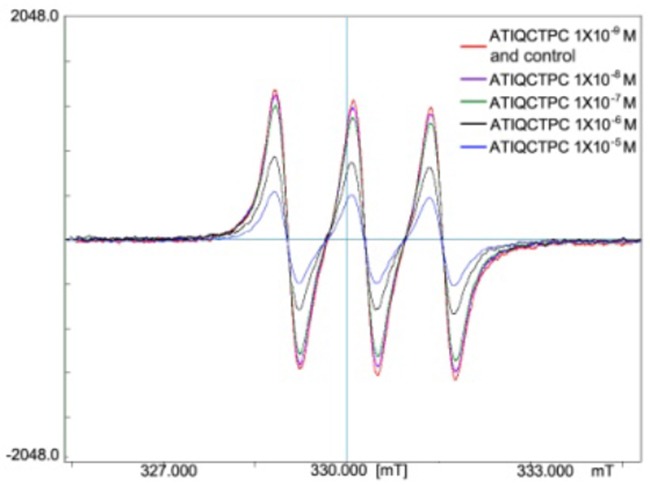
ATIQCTPC concentration-dependently scavenging NO· free radicals, n=6

The effect of NO· free radical and MMP-9 on the inflammation and cancer, as well as the interaction between NO· free radical and MMP-9 have been well known [30]. To explore the contribution of ATIQCTPC scavenging NO· free radical in decreasing serum level of MMP-9, the *in vitro* NO· free radical scavenging assay was performed. Figure 6 indicates that the signal of NO· free radicals is gradually decreased by ATIQCTPC in a concentration (final concentration: 10^−5^ M, 10^−6^ M, 10^−7^ M, 10^−8^ M and 10^−9^ M) dependent manner.

## DISCUSSION

The docking of ATIQCTPC towards the active site of MMP-9 could meet the requirements of a ligand for MMP-9 inhibition [22], and suggests that ATIQCTPC can target MMP-9, thereby lead to the decrease of serum MMP-9 and primary tumor MMP-9 of LLC sarcoma implanted C57BL/6 mice orally treated with ATIQCTPC (0.01 μmol/kg/day) for 11 days. This targeting action benefits ATIQCTPC to inhibit the migration and the invasion of A549 and LLC cells *in vitro*. Furthermore this targeting action benefits ATIQCTPC to decrease the number of lung metastasis nodules of LLC sarcoma implanted C57BL/6 mice in particular. Surprisingly, the 11-day treatment of oral ATIQCTPC (0.01 μmol/kg/day) could effectively decrease the weight and volume of the primary tumor of LLC sarcoma implanted C57BL/6 mice. These findings indicate that ATIQCTPC possesses dual inhibitions of tumor growth and tumor metastasis.

The effect of inflammation on tumor progression and metastasis has been widely demonstrated [31–35]. To understand the anti-tumor and anti-metastasis dual actions of ATIQCTPC the anti-inflammation evaluations were performed. On xylene-induced ear edema mouse model 0.01 μmol/kg ATIQCTPC effectively inhibits ear edema of the mice. This observation explains the reason of 0.01 μmol/kg ATIQCTPC possessing dual inhibitions of tumor growth and tumor metastasis. Besides, 0.01 μmol/kg ATIQCTPC also effectively decreases the serum levels of TNF-α and IL-8 of the inflammatory mice. These observations emphasize the relationships of MMP-9, TNF-α, IL-8 and inflammatory [26–29]. In the pathology of the inflammation and the cancer NO· free radicals and MMP-9 are usually higher [30]. The concentration dependent scavenging of NO· free radicals gives another understanding of ATIQCTPC having dual inhibitions of tumor growth and tumor metastasis. The concentration dependent scavenging of NO· free radicals also implys that via decreasing the level of NO· free radicals ATIQCTPC contributes to the inhibition of MMP-9.

In respect of the *in vivo* benefit of ATIQCTPC inhibiting MMP-9, the activity profiles are the solid evidences. On LLC sarcoma implanted C57BL/6 mouse model the activities of ATIQCTPC in the inhibition of the lung metastasis of LLC and in the slowing of the primary tumor growth are 100-fold higher than those ofATIQC, and the parent compound of ATIQCTPC. On xylene-induced ear edema mouse model the anti-inflammation activity of ATIQCTPC is 100-fold higher than those of ATIQC and aspirin.

Even though the minimal effective dose of ATIQCTPC to inhibit the lung metastasis of LLC, to slow the primary tumor growth, and to block xylene induced ear edema is low to 0.01 μmol/kg, at the dose of 1 μmol/kg ATIQCTPC does not injure the kidney, the liver and the heart of the inflammation mice. Therefore ATIQCTPC therapy is safe enough.

## MATERIALS AND METHODS

### General

C57BL/6 mice and ICR mice were purchased from the Animal Center of Capital Medical University. Work performed was based on a protocol reviewed and approved by the ethics committee of Capital Medical University. The committee assures that the welfare of the mice was maintained in accordance with the requirements of the Animal Welfare Act. Statistical analyses of all the biological data were carried out by use of analysis of variance. *P*-values<0.05 were considered statistically significant. The amino acids (L-configuration), and sodium citrate (analytically pure) were available commercially (Sigma-Aldrich Corp, St Louis MO, USA), acetonitrile spectroscopically pure was available commercially (Thermo Fisher Scientific, Waltham, MA, USA). ATIQCTPC was prepared by following the route depicted in Supplementary Figure 1 of Supplementary Materials. The procedures and the physical/chemical data of the intermediates and ATIQCTPC were also provided in Supplementary Materials. According to Supplementary Materials the HPLC purity of ATIQCTPC is more than 99%. And the IC50 values for 6 cell lines were listed in Supplementary Table 1.

### Cell migration assay

Transwell chamber with cell permeable membrane was used, and A549 and LLC cell migration assays were performed by following a standard method [36]. In brief, A549 cells (5×10^4^ cells/chamber) and serum-free medium in the upper chamber were treated with ATIQC (20 μM) or ATIQCTPC (20 μM). The medium with 10% FBS, the chemotactic factor, was added into the down chamber (600 μL/chamber), the transwell chamber was incubated at 37°C for 6 h, and A549 cells that did not penetrate the membrane and at the bottom of the upper chamber were removed with cotton swabs. A549 cells were fixed with PBS containing 4% formaldehyde for 30 min and stained with 0.1% crystal violet for 15 min. A549 cells that penetrated through the membrane into the down chamber were counted under a light microscope at ×200 to take the count of the cells from 9 visual fields.

### Cell invasion assay

Transwell chamber with cell permeable membrane was used, and A549 and LLC cell migration assays were performed by following a standard method [31]. Membrane at the bottom of each chamber was coated with 50 μL matrigel and air-dried overnight. The chamber was blocked with bovine serum albumin (BSA, 2%, 50μL/chamber), incubated at 37°C for 2 h, and rinsed with PBS. A549 cells (2×10^4^ cells/chamber) and serum-free medium in the upper chamber were treated with ATIQC (20 μM) or ATIQCTPC (20 μM). Into the down chamber, 600 μL of FBS (10%) was added. A549 cells were cultured at 37°C for 24 h and A549 cells that did not penetrate the membrane were removed with cotton swabs. The cells penetrated through the membrane were fixed with 4% formaldehyde for 30 min and stained with 0.1% crystal violet for 10 min. A549 cells that penetrated through the matrigel were counted under a light microscope at ×200 to take the count of the cells from 9 visual fields.

### *In vivo* LLC sarcoma growth and metastasis inhibition assay

Male C57BL/6 mice were maintained at 21°C with a natural day/night cycle in a conventional animal colony. C57BL/6 mice were 10-week old at the beginning of the assay. LLC cells were subcutaneously injected to form solid tumors. To initiate subcutaneous tumors, LLC cells obtained in homogenates of sarcoma form tumor-bearing mice were serially transplanted once per 2 weeks. Subcutaneous tumors were implanted under the skin at the right armpit by injecting 0.2 mL NS containing 1×10^7^ viable tumor cells. Ten days after implantation mice were randomly divided into treatment groups (10 per group) and treated with orally dosed ATIQCTPC (0.01 μmol/kg) or ATIQC (1 μmol/kg) or NS (vehicle) every day for 11 days. C57BL/6 mice were weighed daily and the tumor volume was measured every day. Twenty-four hours after the last administration, the tumor volume was measured and calculated with Length×Width×Width/2^3^, mice were weighed, sacrificed by ether anesthesia, and dissected to immediately obtain and weigh the tumors, and the lungs were also removed and visually examined for the occurrence of tumor metastasis and the numbers of metastatic tumor nodules.

### MMP-9 in the primary tumor and the serum of LLC sarcoma implanted C57BL/6 mice

The primary tumor and the blood of C57BL/6 mice receiving *in vivo* sarcoma growth and metastasis inhibition assay were homogenized and collected, respectively. Then they were centrifuged at 500 g for 10 min to get the primary tumor and serum samples. The MMP-9 in the primary tumor and the serum of the mice treated with ATIQCTPC (0.01 μmol/kg) or ATIQC (1 μmol/kg) or NS (vehicle) was measured according to the guidance of the kits (Mouse Total MMP-9ELISA kit, R&D Systems, Inc., USA).

### *In vivo* anti-inflammatory assay

Male ICR mice weighing 25±2 g were housed in a 12/12 light/dark cycle at a room temperature of 21±2°C for 2 days before use. Food and tap water were supplied ad libitum. The mice were randomly divided into 7 groups of 12 mice, and received ATIQCTPC (0.001, 0.01 and 1 μmol/kg) or ATIQC (1 μmol/kg) or aspirin (110 and 1 μmol/kg) or NS. Thirty minutes later, 0.03 mL of xylene was applied to both the anterior and posterior surfaces of the right ear. The left ear was a control. Two hours after xylene application, the mice were weighed, sacrificed by ether anesthesia, and both ears were removed. With a rubber plug punch of 7 mm aperture the circular sections were taken from the ears for weigh. The ear edema induced by xylene irritant was obtained through subtracting the weight of xylene untreated left ear section from that of xylene treated right ear section.

### Measuring the TNF-a and IL-8 in the serum of ear edema mice

Into an Eppendorf tube containing 50 μL aqueous sodium citrate (3.8%), 450 μL of blood were collected from the inflammatory mice orally receiving NS or 0.01 μmol/kg of ATIQCTPC, and centrifuged at 200 g for 20 min to get the serum. Then, TNF-α and IL-8 in serum were measured according to the guidance of the kits (Mouse TNF-α ELISA kit, Mouse IL-8 ELISA kit, Xitang Biotechnology Co., Shanghai, People's Republic of China).

### Liver and kidney injury assay for inflammatory mice

Into an Eppendorf tube containing 50 μL aqueous sodium citrate (3.8%), 450 μL of blood was collected from the inflammatory mice orally receiving NS or 1 μmol/kg ATIQCTPC and centrifuged at 500 g for 10 min to get serum samples. Then, alanine transaminase (ALT), aspartate transaminase (AST) and creatinine (Cr) in serum were measured according to guidance of the kits (AST/GOT testing kit, ALT/GPT testing kit, Cr testing kit; JCBIO Co., Nanjing, People's Republic of China).

### NO· free radical scavenging assay

NO· free radicals were produced by the reaction of 5 μL solution of 7.325 mg N-methyl-D-glucamine dithiocarbamate (MGD, Sigma) in 1 mL ultrapure water (25 mM), 5 μL solution of 3.475 g FeSO_4_·7H_2_O (Sinopharm Chemical Reagent Beijing Co., Ltd) in 1 mL ultrapure water (12.5 mM), 5 μL solution of 0.25 mg of S-nitroso-N-acetyl-DL-penicillamine (SNAP, Sigma) in 1 mL ultrapure water (1.1 μM), and the signal was recorded on JEOL JES300 ESR as a control of NO· signal (BHNO). The effect of ATIQCTPC on NO· free radicals was defined by comparing the signal of NO· free radicals formed from the reaction of 5 μL solution of 7.325 mg of MGD in 1 mL ultrapure water (25 mM), 5 μL solution of 3.475 g FeSO_4_·7H_2_O in 1 mL ultrapure water (12.5 mM), 5 μL solution of 0.25 mg SNAP in 1 mL ultrapure water (1.1 μM) and 5 μL solution of ATIQCTPC in 1 mL ultrapure water (final concentration: 10^−5^, 10^−4^, 10^−3^ M) with the control of NO· signal.

## SUPPLEMENTARY MATERIALS FIGURES AND TABLES


