# Supplementary material for: ATIQCTPC targeting MMP-9: a key step to slowing primary tumor growth and inhibiting metastasis of lewis lung carcinoma in vivo
Source: Oncotarget. 2017 Jul 10;8(38):63881–9. doi: 10.18632/oncotarget.19172 (PMC5609969; doi:10.18632/oncotarget.19172)
Supplement: Supplementary file 1 [file oncotarget-08-63881-s001.pdf]

ATIQCTPC targeting MMP-9: a key step to slowing primary tumor growth and inhibiting metastasis of lewis lung carcinoma *in vivo*

SUPPLEMENTARY MATERIALS

Synthesis of ATIQCTPC

The preparation of ATIQCTPC was carried out according to scheme 1. The route consists of seven-step reactions.

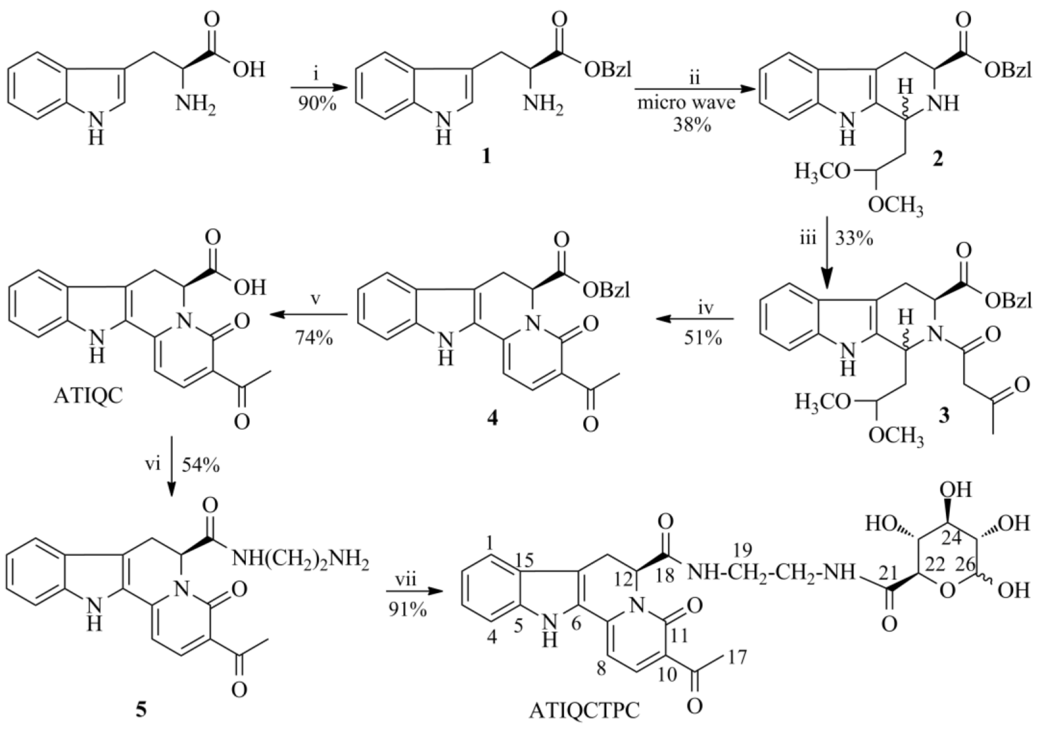

Scheme S1: Synthetic route of ATIQCTPC

Notes:i) Polyphosphoric acid and benzyl alcohol, 80 ºC; ii) CH_2Cl_2, 1,1,3,3- tetramethoxypropxypropane and TFA; iii) CH_3COCH_3, diketene and triethylamine; iv) CH_3COCH_3 and hydrochloric acid (2 M); v) MeOH and aqueous NaOH (2 M); vi) Boc-NH-CH_2-CH_2-NH_2, DCC, HOBt, NMM and THF; vii) Hydrogen chloride in ethyl acetate (4 M); viii) D-Glucuronic acid, DCC, HOBt, NMM and DMF.

Preparing benzyl (S)-2-amino-3-(1H-indol-3-yl) propanoate (1)

In to a mixture of 20.40 g (100.0 mmol) L-Trp and 13.00 g (120.0 mmol) of benzyl alcohol 40.60 g (120.0 mmol) polyphosphoric acid was added. The reaction mixture was at 80 ºC heated for 72 h, cooled to room temperature, into which 30 mL of anhydrous ether was added and stirred for 2 h to collect 35.40 g (90%) of the title compound as colorless solid.

Preparing benzyl (3S)-1-(2,2-dimethoxyethyl)-2,3,4,9-tetrahydro-1H-pyrido- [3,4-b]indole-3-carboxylate (2)

A solution of 8 mL of dichloromethane, 123 mL (48.8 mmol) of 1,1,3,3-tetramethoxypropane, 10.00 g (25.5 mmol) of benzyl (S)-2-amino-3-(1H-indol-3-yl)propanoate (**1**) and 9 mL of trifluoroacetic acid was heated at 35 ºC for 40 min in a microwave reactor. At 0 ºC into the reaction mixture saturated aqueous sodium bicarbonate was slowly

added to adjust pH 7. The organic phase was separated, successively washed with 5% aqueous sodium bicarbonate and saturated aqueous sodium chloride, and dried with anhydrous sodium sulfate. After filtration the filtrate was evaporated under vacuum and the residue was purified on silica gel column (petroleum ether/acetone, 3/1) to give 3.82 g (38%) of the title compound as colorless syrupy. ESI-MS (m/e): 395 $[M + H]^+$. ^{1}H NMR (300 MHz, DMSO-d_6): δ/ppm = 10.83 (d, J = 14.7 Hz, 1H), 7.43~7.26 (m, 7H), 7.03 (t, J = 7.2 Hz, 1H), 6.95 (t, J = 7.5 Hz, 1H), 5.23 (s, 1H), 5.15 (s, 1H), 4.70 (m, 1H), 4.26 (d, J = 10.2 Hz, 1H), 4.17 (d, J = 7.5 Hz, 1H), 3.97 (t, J = 5.7 Hz, 1H), 3.76 (d, J = 7.5 Hz, 1H), 3.36~3.23 (m, 6H), 3.03 - 2.65 (m, 2H), 2.42 (m, 1H), 2.13 (m, 1H), 1.82 (m, 1H).

Preparing benzyl (3S)-1-(2,2-dimethoxyethyl)-2-(3-oxobutanoyl)-2,3,4,9-tetra- hydro-1H-pyrido[3,4-b]indole-3-carboxylate (3)

At 0 ºC and with stirring to a solution of 3.82 g (9.7 mmol) benzyl (3S)-1-(2,2- dimethoxyethyl)-2,3,4,9-tetrahydro-1H-pyrido[3,4-b]indole-3-carboxylate (**2**) in 50 mL of acetone 1.2 mL of diketene and 0.8 mL of triethylamine were added, and stirred at room temperature for 17 h. To this reaction mixture 2.5 mL of distilled water was added and stirred at 0 ºC for 1 h. The solution was evaporated under vacuum, and the residue was extracted with dichloromethane for three times. The organic phase was successively washed with 5% aqueous sodium bicarbonate and saturated aqueous sodium chloride, and dried with anhydrous sodium sulfate. After filtration the filtrate was evaporated under vacuum and the residue was purified on silica gel column (petroleum ether/acetone, 4/1) to give 1.53 g (33%) of the title compound as colorless syrupy. ESI-MS (m/e): 479 $[M + H]^+$. ^{1}H NMR (300 MHz, DMSO-d_6): δ/ppm = 10.84 (d, J = 3.6 Hz, 1H), 7.53~7.34 (m, 3H), 7.33 - 7.25 (m, 4H), 7.04~7.01 (m, 2H), 5.24~5.19 (m, 2H), 4.96 (m, 1H), 4.61 (m, 1H), 4.05 (m, 1H), 3.32~3.23 (m, 6H), 3.15 (s, 2H), 3.00~2.93 (m, 2H), 2.16 (d, J = 1.5 Hz, 3H), 1.97 (m, 1H), 1.83 (m, 1H).

Preparing benzyl (S)-3-acetyl-4-oxo-4,6,7,12-tetrahydroindolo[2,3-a]quinolizine-6-carboxy late (4)

At 0 ºC and with stirring to a solution of 858 mg (1.8 mmol) of benzyl (3S)-1-(2,2-dimethoxyethyl)-2-(3-oxobutanoyl)-2,3,4,9-tetrahydro-1H-pyrido[3,4-b] indole-3-carboxylate (**3**) in 22 mL of acetone 0.3 mL of hydrochloric acid (2 M) was added, and the reaction mixture was stirred at room temperature for 24 h, to which aqueous sodium bicarbonate (5%) was slowly added to adjust pH 7. The solution was evaporated under vacuum, the residue was extracted with ethyl acetate for three times, and successively washed with aqueous sodium bicarbonate (5%), citric acid (5%) and saturated aqueous sodium chloride. The organic phase was dried with anhydrous sodium sulfate. After filtration the filtrate was evaporated under vacuum and the residue was purified on silica gel column (petroleum ether/acetone, 4/1) to give 377 mg (51%) of the title compound as yellow powder. ESI-MS (m/e): 413 $[M + H]^+$. ^{1}H NMR (300 MHz, DMSO-d_6): δ/ppm = 11.99 (s, 1H), 8.21 (d, J = 7.5 Hz, 1H), 7.23~7.03 (m, 9H), 6.90 (d, J = 7.8 Hz, 1H), 6.19 (d, J = 6.0 Hz, 1H), 5.10 (d, J = 4.8 Hz, 2H), 3.44 (dd, J_1 = 17.4 Hz, J_2 = 7.5 Hz, 2H), 2.58 (s, 3H). ^{13}C-NMR (75 MHz, DMSO-d_6): δ/ppm = 196.24, 170.05, 160.99, 143.43, 142.90, 139.54, 136.01, 132.04, 131.61, 128.25, 127.27, 127.19, 127.15, 125.75, 125.45, 123.85, 120.76, 120.51, 113.71, 112.69, 100.58, 67.03, 51.11, 31.07, 22.86.

Preparing (S)-3-acetyl-4-oxo-4,6,7,12-tetrahydroindolo[2,3-a]quinolizine-6-carboxylic acid (ATIQC)

At 0 ºC and with stirring to a solution of 380 mg (0.9 mmol) of benzyl (S)-3-acetyl-4-oxo-4,6,7,12-tetrahydroindolo[2,3-a]quinolizine-6-carboxylate (**4**) in 15 mL of methanol aqueous NaOH (2 M) was slowly added to adjust pH 12. This mixture was stirred at 0 ºC for 96 h, and saturated $KHSO_4$ aqueous solution was slowly added to adjust pH 7. The reaction mixture was evaporated under vacuum, and the residue was dissolved in 5 mL of distilled water, to which saturated aqueous $KHSO_4$ was at 0 ºC slowly added and stirred to adjust pH value to 3. The reaction mixture was filtered and the collected yellow powder was rinsed with distilled water to give 220 mg (74%) of the title compound as yellow powder. Mp: 205~207 ºC; $[\alpha]_D^{25}$= -28.3 (c = 0.10, CH_3OH); IR (KBr, cm^{-1}): 3319, 3061, 2291, 2927, 2586, 1743, 1656, 1587, 1546, 1496, 1438, 1425, 1363, 1330, 1284, 1236, 1201, 1145, 1111, 1029, 972, 852, 781, 746, 624, 567 cm^{-1}; ESI-MS (m/e): 321 $[M-H]^-$; ^{1}H NMR (500 MHz, DMSO-d_6): δ/ppm = 11.92 (s, 1H), 8.17 (d, J = 8.0 Hz, 1H), 7.67 (d, J = 7.0 Hz, 1H), 7.44 (d, J = 10.0 Hz, 1H), 7.28 (t, J = 6.0 Hz, 1H), 7.09 (t, J = 6.0 Hz, 1H), 6.85 (d, J = 8.0 Hz, 1H), 5.96 (d, J = 6.0 Hz, 1H), 3.73 (d, J = 16.0 Hz, 1H), 2.55 (s, 3H). ^{13}C-NMR (75 MHz, DMSO-d_6): δ/ppm = 196.33, 171.58, 160.99, 143.20, 143.05, 139.47, 127.26, 125.66, 125.46, 123.73, 120.69, 120.46, 113.94, 112.68, 100.38, 52.37, 31.11, 22.74.

Preparing (S)-(2-(3-acetyl-4-oxo-4,6,7,12-tetrahydroindolo[2,3-a]quinolizine-6-carboxamido)ethyl)carbamate (5)

At 0 °C to a solution of 0.5 mL (4.5 mmol) of N-methylmorpholine, 800 mg (5.0 mmol) of

$NHCH_2CH_2NH_2$,865 mg (4.2 mmol) of DCC, 473 mg (3.5 mmol) of HOBt and 16 mL of anhydrous tetrahydrofuran (THF) a solution of1.13 g (3.5 mmol) of ATIQC in 16 mL of anhydrous THF was slowly added. This mixture was stirred at 0 °C for 1 h, at room temperature for 5 h, and thin-layer chromatography indicated the complete disappearance of ATIQC. The reaction mixture was filtered and the filtrate was evaporated under vacuum. The residue was dissolved in 150 mL of ethyl acetate and the solution was successively washed with aqueous sodium bicarbonate (5%) and saturated aqueous sodium chloride, the ethyl acetate phase was dried with anhydrous sodium sulfate. After filtration and evaporation under vacuum, the residue was purified on silica gel column (CH_2Cl_2/CH_3OH, 30/1) to give 571 mg (54%) of the title compound as yellow powder. ESI-MS (m/e): 363 $[M - H]^-$.^{1}H NMR (300 MHz, DMSO-d_6):δ/ppm = 11.94 (s, 1H), 8.59 (t, *J* = 5.4 Hz, 1H), 8.18 (d, *J* = 7.8 Hz, 1H), 7.83 (s, 3H), 7.62 (d, *J* = 8.1 Hz, 1H), 7.44 (d, *J* = 8.1 Hz, 1H), 7.28 (t, *J* = 7.2 Hz, 1H), 7.10 (t, *J* = 7.2 Hz, 1H), 6.88 (d, *J* = 7.8 Hz, 1H), 5.95 (d, *J* = 6.9 Hz, 1H), 3.74 (d, *J* = 17.4 Hz, 1H), 3.24~3.18(m, 2H), 2.76 (t, *J* = 6.6 Hz, 2H), 2.56 (s, 3H). ^{13}C-NMR (75 MHz, DMSO-d_6): δ/ppm = 196.34, 169.90, 161.07, 143.74, 143.07, 139.39, 127.50, 125.54, 125.42, 123.67, 120.53, 120.34, 113.25, 112.63, 100.37, 53.00, 38.67, 37.31, 31.10, 23.71.

Preparing (6S)-3-acetyl-4-oxo-N-(2-(3,4,5,6-tetrahydroxytetrahydro-2H-pyran- 2-carboxamido)ethyl)-4,6,7,12-tetrahydroindolo[2,3-a] quinolizine-6-carboxamide (ATIQCTPC)

At 0 °C to a solution of 0.2 mL (1.8 mmol) of N-methylmorpholine, 182 mg (0.5 mmol) of (S)-3-acetyl-N-(2-aminoethyl)-4-oxo-4,6,7,12-tetrahydroindolo[2,3-a] quinolizine-6-carboxamide (**5**),124 mg (0.6 mmol) of DCC, 68 mg (0.5 mmol) of HOBt, 16 mL of anhydrous DMF and 388 mg (2.0 mmol) D-glucuronic acid were added, at 0 °C for stirred for 1 h, at room temperature stirred for 5 h and thin-layer chromatography indicated the complete disappearance of (**6**). At 40 °C the reaction mixture was evaporated under vacuum to remove anhydrous DMF, and the residue was purified by C18 column chromatography (acetonitrile/H_2O, 2/8) to give 46 mg (91%) of the title compound as yellow powders. Mp: 171~172 ºC; $[\alpha]_D^{25}$= +8.4 (c = 0.10, H_2O); IR (KBr, cm^{-1}): 3410, 3251, 1643, 1637, 1614, 1587, 1552, 1504, 1384, 1361, 1332, 1284, 1263, 1246, 1114; HRMS-ESI(-) calcd for $C_{26}H_{27}N_4O_9$: 539.1784, found: 539.1777; ^{1}H NMR (800 MHz, DMSO-d_6): δ/ppm = 11.87 (s, 1H), 8.32 (m, 1H), 8.17 (d, *J* = 8.0 Hz, 1H), 7.94 (m, 1H), 7.63 (t, *J* = 6.4 Hz, 1H), 7.43 (d, *J* = 8.0 Hz, 1H), 7.28 (t, *J* = 8.0 Hz, 1H), 7.10 (t, *J* = 8.0 Hz, 1H), 6.85 (d, *J* = 7.2 Hz, 2H), 6.50 (s, 1H), 5.91 (d, *J* = 7.2 Hz, 1H), 5.03 (s, 1H), 4.95 (m, 1H), 4.80 (s, 1H), 4.60 (s, 1H), 4.30 (m, 1 H), 3.63 (m, *J* = 16.8 Hz, 1H), 3.48 (m, 1H), 3.40(m, 1H), 3.32 (m, 1H), 3.17 (m, 1H), 3.08~2.95 (m, 5H), 2.56 (s, 3H). ^{13}C-NMR (200 MHz, DMSO-d_6): δ/ppm = 196.37, 170.67, 169.77, 169.73, 169.28, 161.05, 143.85, 143.03, 139.36, 127.54, 125.59, 125.38, 123.59, 120.55, 120.40, 120.35, 119.94, 113.29, 113.28, 112.59, 100.28, 97.76, 93.22, 76.74, 75.84, 74.88, 73.11, 72.75, 72.33, 72.13, 52.91, 39.15, 38.34, 31.17, 23.89.

Method for determining HPLC purity of ATIQCTPC

An Agilent Technologies 1200 Series HLPC system (Agilent Technologies, Santa Clara, CA, USA) was used to determine the purity of ATIQCTPC. The sample was loaded on a Waters XTerra C18 reversed-phase column (2.1×150 mm, 5 μm; Waters Limited, Hertfordshire, UK) protected by a guard column of the same material (5×10 mm, 5 μm). The column thermostat was maintained at 40 °C. To the column, 5 μL of a solution of ATIQCTPC in methanol was injected for analysis. The mobile phase consisted of aqueous acetonitrile (20%). The flow rate was 0.2 mL/minute. The column was washed with aqueous acetonitrile (20%), and equilibrated to initial conditions for 15 minutes. Ultraviolet (UV) absorption spectra were recorded online. The UV detector was set to a scanning range of 200~500 nm, and a wavelength of 431 nm was used to monitor ATIQCTPC. The chromatogram was recorded, which gave ATIQCTPC three peaks at 17.70, 20.23 and 21.29 minutes and total purity of 98.90%. According to LC-ESI-FTMS three peaks share the same mass of 539.178 $[M-H]^-$, which are from the terminal isomers α, β and open-chain forms of D-glucuronic acid ring.

MTT assay of ATIQCTPC

K562, A549, LLC, S180, LO2 and HaCaT cells were treated with ATIQC, ATIQCTPC and doxorubicin (Dox) at a series of concentrations (0.1~150 μM) for 72 h in a 96-well plate. MTT was added to a final concentration of 0.5 mg/mL and further incubated for 4 h. The yellow-colored MTT was reduced to insoluble purple-colored formazan in living cells. The culture medium was removed and DMSO was added to dissolve formazan. The absorbance of the formazan containing solution was then measured at 570 nm using a Thermo MULTISJKAN MK3 spectrophotometer. The IC_{50} values of ATIQC and ATIQCTPC against the proliferation of K562, A549, LLC, S180, LO2 and HaCaT cells are

shown in Table S1. As seen the IC_{50} values of ATIQC and ATIQCTPC against cancer cell proliferation are more than 100 μM. Therefore ATIQC and ATIQCTPC exhibit no cytotoxicity.

Molecular docking

The 3D structure of ATIQCTPC was built using a 3D-sketcher module and energy minimized in Discovery Studio 4.0 with a SMART minimizer using the CHARMm force field. The crystal structure of MMP-9 (PDB ID: 5I12) was from the Protein Data Bank, and the binding sphere (12.0 Å) of the (2R)-2-[2-[[(2R,3R,4R,5S,6R)-3- acetamido-4,5-diacetyloxy-6-(acetyloxymethyl)oxan-2-yl]carbamothioylamino] ethyl-(4-phenylphenyl)sulfonylamino]-3-methylbutanoic acid (standard ligand) domain was defined. The water molecules were removed, and hydrogen atoms were added under the CHARMm force field. Docking calculations were performed using the LibDock module implemented in Accelrys Discovery studio 4.0, which is a high-through put docking algorithm that positions catalyst generated ligand conformations in the protein active site based on polar and apolar interaction sites (hotspots). The results could be displayed by analyzing and scoring docked ligand poses. To find a top rank pose and measure the goodness of a docking study, the LibDock Score was used as the criteria. The conformation generation of the ligand was carried out using the BEST method. The energy threshold was set to 20 kcal·mol^{-1}, the maximum minimization steps were set to 1000, the minimization RMS gradient was set to 0.001 Å and the maximum generated conformations were set to 255 with a RMSD cut-off of 1.0 Å. The libdock scores of the standard ligand, ATIQCTPC, LU8C-FP, TAIII, BHIMHA and (6S)-6 are shown in Table S2.

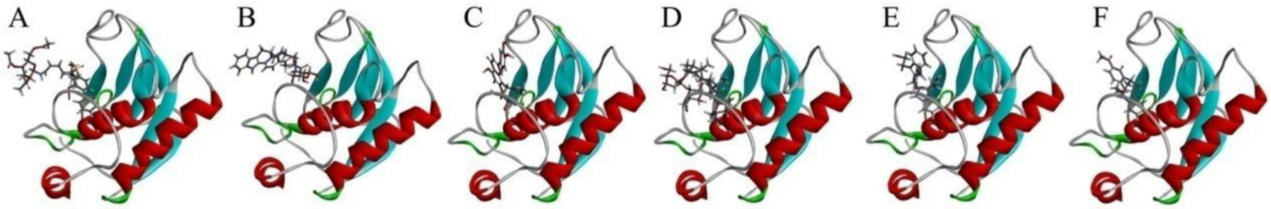

Supplementary Figure 1: Docking feature of 6 compounds in the active site of MMP-9. **(A)** Docking feature of the standard ligand ((2R)-2-[2-[[(2R,3R,4R,5S,6R)-3- acetamido-4,5-diacetyloxy-6-(acetyloxymethyl)oxan-2-yl]carbamothioylamino]ethyl-(4-phenylphenyl)sulfonylamino]-3-methylbutanoic acid) in the active site of MMP-9. **(B)** Docking feature of ATIQCTPC in the active site of MMP-9. **(C)** Docking feature of LU8C-FP in the active site of MMP-9. **(D)** Docking feature of TAIII in the active site of MMP-9. **(E)** Docking feature of BHIMHA in the active site of MMP-9. **(F)** Docking feature of (6S)-6 in the active site of MMP-9.

Supplementary Table 1: IC_{50} values of ATIQC and ATIQCTPC against cancer cell proliferation

Compound	IC_{50} values (Mean ± SD µM) for the following cancer cells					
	K562	A549	LLC	S180	LO2	HaCaT
Dox	0.88 ± 0.07	1.37 ± 0.29	0.87 ± 0.21	0.71 ± 0.11	0.93 ± 0.12	0.56 ± 0.09
ATIQC	> 100	> 100	> 100	> 100	> 100	> 100
ATIQCTPC	> 100	> 100	> 100	> 100	> 100	> 100

Note: n=6.

Supplementary Table 2: Libdock scores of 5 compounds in figure 1 and the standard ligand

Compound	standard ligand	ATIQCTPC	LU8C-FP	TAIII	BHIMHA	(6S)-6
Libdock score	95.08	118.02	110.29	109.02	107.23	106.80
